# Molecular characterization of field infectious bursal disease virus isolates from Nigeria

**DOI:** 10.14202/vetworld.2016.1420-1428

**Published:** 2016-12-15

**Authors:** Ijeoma O. Nwagbo, Ismaila Shittu, Chika I. Nwosuh, George O. Ezeifeka, Frederick J. C. Odibo, Linda O. Michel, Daral J. Jackwood

**Affiliations:** 1 Department of Virology, Viral Research Division, National Veterinary Research Institute, Vom, Plateau State, Nigeria; 2 Department of Veterinary Microbiology and Parasitology, College of Veterinary Medicine, Micheal Okpara University of Agriculture Umudike, Abia State, Nigeria; 3 Department of Applied Microbiology and Brewing, Faculty of Biosciences. Nnamdi Azikiwe University Awka, Anambra State, Nigeria; 4 Department of Veterinary Preventive Medicine, Food Animal Health Research Program, Ohio Agricultural Research and Development Center, The Ohio State University, 1680 Madison Avenue, Wooster, OH 44691, USA

**Keywords:** infectious bursal disease virus, Nigeria, novel, reassortant, very virulent

## Abstract

**Aim:**

To characterize field isolates of infectious bursal disease virus (IBDV) from outbreaks in nine states in Nigeria through reverse transcription polymerase chain reaction (RT-PCR) and sequence analysis of portions of the VP2 and VP1 genes and to determine the presence or absence of reassortant viruses.

**Materials and Methods:**

A total of 377 bursa samples were collected from 201 suspected IBD outbreaks during 2009 to 2014 from nine states in Nigeria. Samples were subjected to RT-PCR using VP2 and VP1 gene specific primers, and the resulting PCR products were sequenced.

**Results:**

A total of 143 samples were positive for IBDV by RT-PCR. These assays amplified a 743 bp fragment from nt 701 to 1444 in the IBDV VP2 hypervariable region (hvVP2) of segment A and a 722 bp fragment from nt 168 to 889 in the VP1 gene of segment B. RT-PCR products were sequenced, aligned and compared with reference IBDV sequences obtained from GenBank. All but one hvVP2 sequence showed similarity to very virulent IBDV (vvIBDV) reference strains, yet only 3 of the VP1 67 VP1 sequences showed similarity to the VP1 gene of vvIBDV. Phylogenetic analysis revealed a new lineage of Nigerian reassortant IBDV strains.

**Conclusion:**

Phylogenetic analysis of partial sequences of genome segment A and B of IBDV in Nigeria confirmed the existence of vvIBDV in Nigeria. In addition, we noted the existence of reassortant IBDV strains with novel triplet amino acid motifs at positions 145, 146 and 147 in the reassorted Nigerian IBDV.

## Introduction

Infectious bursal disease virus (IBDV) is a bi-segmented dsRNA virus that is a member of the family *Birnaviridae* and genus *Avibirnavirus* [1]. It is the causative agent of IBD, a highly contagious immunosuppressive disease [2]. Strains of IBDV can be grouped within two distinct serotypes: Serotype 1 is pathogenic in chickens and serotype 2, isolated from turkeys, is not pathogenic in chickens or turkeys [3]. Serotype 1 strains are further classified into very virulent (vv), classical virulent, and sub-clinical on the basis of pathogenicity. The viruses are generally classified into classic and variant antigenic strains, but antigenic drift has made these designations outmoded [4].

The viral genome of IBDV has two double-stranded RNA segments; A is approximately 3.2 kb and B is approximately 2.8 kb [3]. Segment A contains partially overlapping open reading frames (ORFs), ORF1 and ORF2. The small ORF1 encodes a non-structural protein VP5 (17 kDa), whereas the large ORF2 encodes a precursor polyprotein (NH2-VP2-VP4-VP3-COOH), which is cleaved by autoproteolysis to produce the viral capsid protein VP2 (38 kDa), the ribonucleoprotein VP3 (33 kDa) and the viral protease VP4 (29 kDa) [1]. Segment B contains one ORF encoding VP1 (91 kDa), an RNA-dependent RNA polymerase that is responsible for viral genome replication and mRNA synthesis [1,5]. The VP2 antigens of IBDV display high amino acid variability within a hypervariable region (hvVP2) located from amino acid residues 206-350 [6]. This region is responsible for antigenic variation, tissue-culture adaptation and it is partially responsible for viral virulence [7]. The previous reports suggest that VP1 also contributes to the virulence of IBDV [8,9]. Phylogenetic studies have shown that the genome segment B of vvIBDV is highly conserved and distinct from that of non-vvIBDVs [10] and is responsible for their high mortality phenotype [11]. Reassortment in the genome segments B and amino acid substitution in genome segment A has been shown to occur in vvIBDV [11,12]; therefore, it is necessary to analyze both genome segments in molecular studies to correctly identify these virus strains [11,13,14].

Since it was first reported in Nigeria in 1973, IBDV has acquired an endemic status despite vaccination protocols [15,16], and vvIBDV has been reported throughout the country [17-21]. The aim of this study was to identify and characterize the IBDV strain(s) currently circulating in Nigerian commercial and backyard chicken flocks and to determine if the vvIBDVs identified are true vv strains or if they are reassorted vvIBDVs.

## Materials and Methods

### Ethical approval

This study does not involve use of live animals or human subject.

### Bursa samples

**Figure-1:**
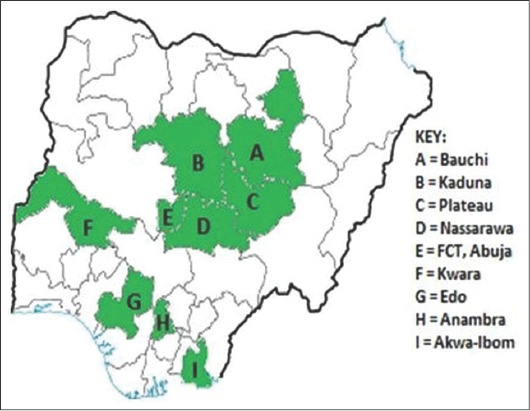
Map of Nigeria showing states (green) of origin of viruses.

We examined 201 outbreaks of IBD that occurred from 2009 to 2014 in vaccinated and unvaccinated commercial and backyard chicken flocks in nine states in Nigeria: Anambra, Edo (Benin), Plateau, Nasarawa, Kaduna, Akwa Ibom (Uyo), Bauchi, Kwara and Abuja (Figure-1). A total of 201 bursae were processed and blotted onto FTA cards (GE Whatman, Sigma-Aldrich).

### Viral RNA extraction and reverse transcription polymerase chain reaction (RT-PCR) amplification

Viral RNA was eluted from FTA cards using 300 µl of TE buffer (10 mM Tris-HCl, pH 8.0, 1 mM ethylenediaminetetraacetic acid). Extraction of RNA was carried out using the standard Trizol (Ambion) procedure described by Jackwood *et al*. [22]. The double-stranded RNA was denatured at 95°C for 5 min before RT-PCR was performed with an AgPath-ID™ One-Step RT-PCR kit (Ambion; Applied Biosystems, Foster City, CA) according to manufacturer’s instructions. Primers 743-F: 5´-GCCCAGAGTCTACACCAT-3´ and 743-R: 5´-CCCGGATTATGTCTTTGA-3´ [23] were used to amplify the hvVP2 region of genome segment A and primers B-168AF (5´-CATAAAGCCTACAGCTGGAC-3´) and B-889R (5´-GTCCACTTGATGACTTGAGG-3´) [24], were used to amplify a portion of VP1. For segment A, RT was performed at 48°C for 30 min and then inactivated at 95°C for 10 min followed by 35 cycles at 95°C for 30 s and 57°C for 90 s. After completion of the 35 cycles, a final extension at 72°C for 5 min was performed. Cycling conditions were the same for segment B, but the annealing temperature was 55°C.

### Sequence analysis

The RT-PCR products were prepared for sequencing using a Wizard SV Gel and PCR Clean-Up System (Promega Corp., Madison, WI). Cycle sequencing was conducted at the University of Wisconsin, Biotechnology Center, DNA Sequence Laboratory (Madison, WI). Nucleotide sequence results were downloaded using Chromas (Technelysium Pty. Ltd., Queensland, Australia). Sequence analysis and Clustal W alignments were conducted using Accelrys Gene v2.5 software (Accelrys, San Diego, CA). Phylogenetic analysis was conducted with MEGA version 6.0 software [25] using neighbor-joining (NJ) and unweighted pair-group methods using arithmetic averages (UPGMA) each with 1000 bootstrap replicates. The nucleotide and deduced amino acid sequences of segments A and B were aligned with previously published IBDV sequences obtained from Genbank. Nucleotide and amino acid sequences obtained from the Nigerian IBDV strains identified in this study were submitted to Genbank.

## Results

A total of 201 bursa samples collected from Nigerian commercial and backyard poultry flocks during 2009-2014 were tested by RT-PCR for the presence of IBDV. A 743 bp fragment of the hvVP2 region was amplified from 143 (71%) of the samples tested. A 722 bp fragment of the VP1 gene was amplified from 86 of these. Nucleotide sequences of 105 of the hvVP2 samples and 67 of the VP1 samples were determined. The Genbank accession numbers for segment A are KP152231-KP152322 and KP266322-KP266334 and for segment B they are KP172084-KP172144 and KP266335-KP266340.

### Sequence analysis of hvVP2

The nucleotide and deduced hvVP2 amino acid sequences of the 105 Nigerian IBDV were aligned and compared with hvVP2 sequences from two reference vvIBDV (UK661 and OKYM), one variant (Del E), as well as strains from previous outbreaks in Nigeria and other countries obtained from GenBank and identified by their accession numbers and country of origin. The results showed 13 viruses detected in this study from three states (Akwa Ibom, Edo, and Plateau) had 100% nucleotide identity with three previously published Nigerian IBDV (Accession numbers JX424071.1, JX424073.1, and KM870808) (data not shown). Of the 105 Nigerian viruses, the VP2 amino acid sequences of 4 were identical to IBDV vaccine strains ABIC and MB. The remaining strains were most closely related with the vvIBDVs (95.0-98.9%) with the exception of Nassarawa77/NG/no2/2013 that was a non-vvIBDV and appeared to be related to classic IBDV strains.

The hvVP2 amino acid sequences of the Nigerian deduced vvIBDV had amino acids generally conserved among the vvIBDV: A222, I242, I256, I294 and S299. The Nassarawa77/NG/no2/2013 virus had A222 but it lacked the virulent amino acid markers typically observed in vvIBDV. It had amino acids H253, N279 and T284 typically seen in attenuated IBDV strains (Table-1). The Nigerian viruses also displayed variations at amino acid 300. Only five Nigerian viruses had E300 which was similar to UK661. Substitutions of amino acids at position 300 (E→A) and (E→Q) were seen in 81 and 19 of the Nigerian viruses, respectively. Substitution of the amino acid at position 300 (E→A/Q) may alter the biological characteristics of IBDVs which could lead to vaccination failure [4].

**Table-1:** Comparison of genome segment A amino acid substitutions at selected positions between Nigerian field viruses and other published strains of IBDV.

Strain/Isolate	Amino acid substitution at positions^A^															

	219	222	242	253	254	256	261	269	272	279	284	294	299	300	315	330
AJ878898 vvIBDV UK661	Q	A	I	Q	G	I	I	T	I	D	A	I	S	E	S	S
UYO192/NG/2014	.	.	.	.	S	.	.	.	.	.	.	.	.	A	.	.
PLATEAU43/NG/2012	.	.	.	.	S	.	.	S	.	.	.	.	.	Q	.	.
PLATEAU160/NG/2014	T	.	.	.	S	.	.	S	.	.	.	.	.	Q	.	.
KWARA148/NG/2014	.	.	.	.	.	.	.	.	T	N	.	.	.	.	.	.
BAUCHI37/NG/2012	.	T	.	.	S	.	.	.	.	.	.	.	.	A	.	.
BENIN35/NG/2012	.	.	.	.	S	.	.	.	.	.	.	.	.	A	.	.
NASSARAWA77/NG/no2/2013	.	.	V	H	S	V	.	.	.	N	T	L	N	.	.	.
NASSARAWA77/NG/2013	.	.	.	L	S	.	.	S	.	.	.	.	.	Q	.	R
PLATEAU78/NG/2011/2	P	.	.	.	S	.	.	S	.	.	.	.	.	Q	.	R
PLATEAU78/NG/2011/1	T	.	.	L	S	.	.	S	.	.	.	.	.	Q	.	.
PLATEAU92/NG/2013	.	.	.	.	S	.	T	.	.	.	.	.	.	A	.	.
PLATEAU75/NG/2011	.	.	.	.	S	.	.		.	.	.	.	.	A	.	.
ANAMBRA50/NG/2012	T	.	.	.	S	.	.	S	.	.	.	.	.	Q	.	.
KWARA176/NG/2014	.	.	.	.	S	.	.	.	.	.	.	.	.	A	T	.
BAA08555 OKYM	.	.	.	.	.	.	.	.	.	.	.	.	.	.	.	.
AJ878905 VARIANT DEL E	.	T	V	.	S	V	.	.	.	N	.	L	N	.	.	.
AAG01816 VARIANT T1	.	T	V	.	N	V	.	.	.	N	.	L	N	.	.	.
D00499 CLASSIC STC	.	P	V	.	.	V	.	.	.	.	.	L	N	.	.	.

A Dots indicate sequences identical to those of the UK661 strain. IBDV=Infectious bursal disease virus

### Sequence analysis of segment B

Partial nucleotide (nt168-889) and deduced amino acid (68-248) sequences of the VP1 gene of 67 Nigerian viruses were aligned and compared with the VP1 sequences of two reference vvIBDV (UK661 and HK46) and one variant (Del E) IBDV strains [11]. Only three out of 67 Nigerian viruses showed high nucleotide sequence identity with vvIBDV strains UK661 and HK46 (99.3%). The VP1 nucleotide sequence identity of the remaining 64 viruses to these vvIBDV strains was only 83.9-85.7%.

Interestingly, the triplet amino acid positions 145, 146 and 147 of VP1 of the Nigerian viruses had motifs not seen in any of the other viruses used for comparison (Table-2). The vvIBDV have the triplet TDN while lower pathogenicity viruses generally have NED at these three positions. The following motifs and the number of times they were observed in the Nigerian viruses were; QEG (n=50), HEG (n=12), QDG (n=1), HDG (n=1) and TDN (n=3).

**Table-2:** Comparison of genome segment B amino acid substitutions at selected positions between Nigerian field viruses and published strains of IBDV.

Strain/isolate	Type^A^	Amino acid substitution at positions^B^													

		96	119	123	141	145	146	147	148	150	154	161	163	219	242
AJ878668_HK46	vv	T	E	A	V	T	D	N	L	D	L	D	A	D	E
D49707_OKYM	vv	.	.	.	.	.	.	.	.	.	.	.	.	.	.
PLATEAU19/NG/2009	Non-vv	.	D	.	I	H	E	G	.	E	.	A	V	E	D
KWARA148/NG/2014	vv	N	.	.	.	.	.	.	.	.	.	.	.	.	.
PLATEAU161/NG/2014	Non-vv	.	D	.	I	Q	E	G	.	E	.	.	V	E	D
UYO167/NG/2014	Non-vv	.	D	.	I	H	E	G	.	E	.	.	V	E	D
PLATEAU39/NG/2012	Non-vv	.	.	.	I	Q	E	G	.	E	.	.	V	E	D
PLATEAU157/NG/2014	Non-vv	.	D	.	I	Q	D	G	.	E	I	.	V	E	D
UYO190/NG/2014	Non-vv	.	.	T	T	Q	E	G	P	E	.	.	V	E	D
KWARA141/NG/2014	vv	N	.	.	.	.	.	.	.	.	.	A	.	.	.
PLATEAU96/NG/2013	Non-vv	.	.	.	I	Q	E	G	P	E	.	.	.	E	D
PLATEAU40/NG/2012	vv	N	.	.	.	.	.	.	.	.	.	A	.	.	.
PLATEAU82/NG/2013	Non-vv	.	.	.	I	Q	E	G	P	E	.	.	.	E	D
PLATEAU90/NG/2013	Non-vv	.	D	.	I	Q	E	G	.	E	I	.	V	E	D
PLATEAU7/NG/2009	Non-vv	.	D	.	I	H	D	G	.	E	.	.	V	E	D
PLATEAU7/NG/2009	Non-vv	.	D	.	I	H	D	G	.	E	.	.	V	E	D
AJ878671_HENAN	vv	.	.	.	.	.	E	G	.	.	.	.	.	.	D
AY099457_T09	vv	.	.	.	.	.	.	.	.	.	.	.	.	.	.
AJ878656_BURS706	Vaccine	.	.	.	.	N	E	G	.	.	.	.	.	.	D
AJ878666_UK661	vv	.	.	.	.	.	.	.	.	.	.	.	.	.	.
AJ878676_DEL_E	Variant	.	.	.	.	N	E	D	.	.	.	.	.	.	D

A Type=The molecular strain of IBDV, Non-vv=Segment B sequence is not typical of a vvIBDV. Vaccine and Variant strains are not vvIBDV,

B Dots indicate sequences identical to those of the HK46 vvIBDV strain. IBDV=Infectious bursal disease virus, vv=Very virulent

### Phylogenetic analysis of hvVP2

**Figure-2:**
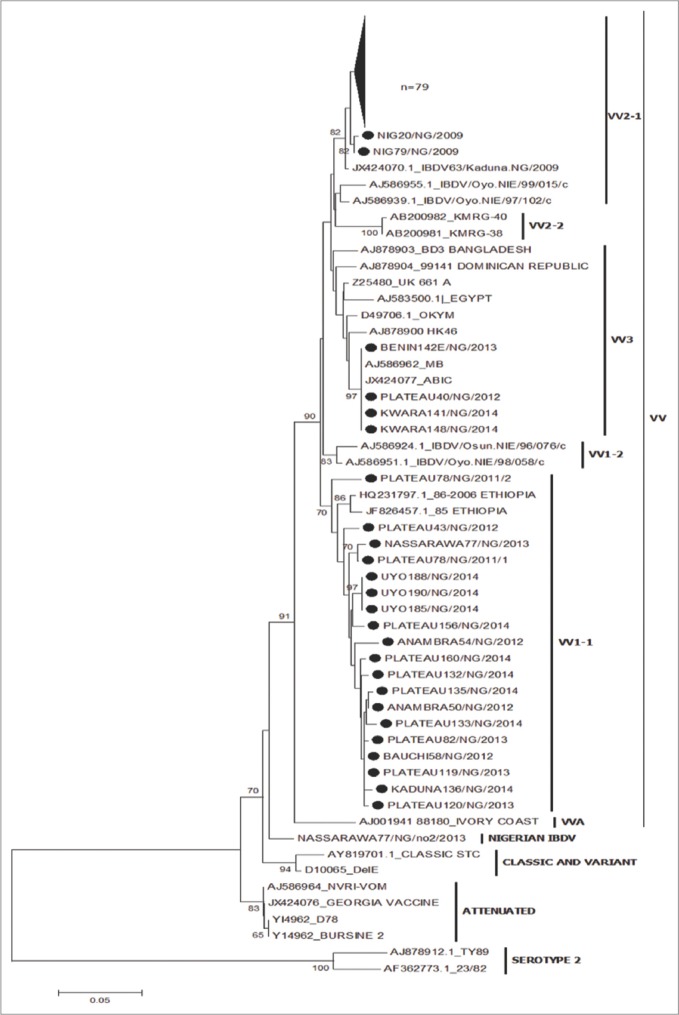
Phylogenetic analysis of the VP2 hypervariable region nucleotide sequences of Nigerian infectious bursal disease virus (IBDVs) and other IBDV strains from Genbank using the neighbor joining method with 1000 bootstrap replication.

**Figure-3:**
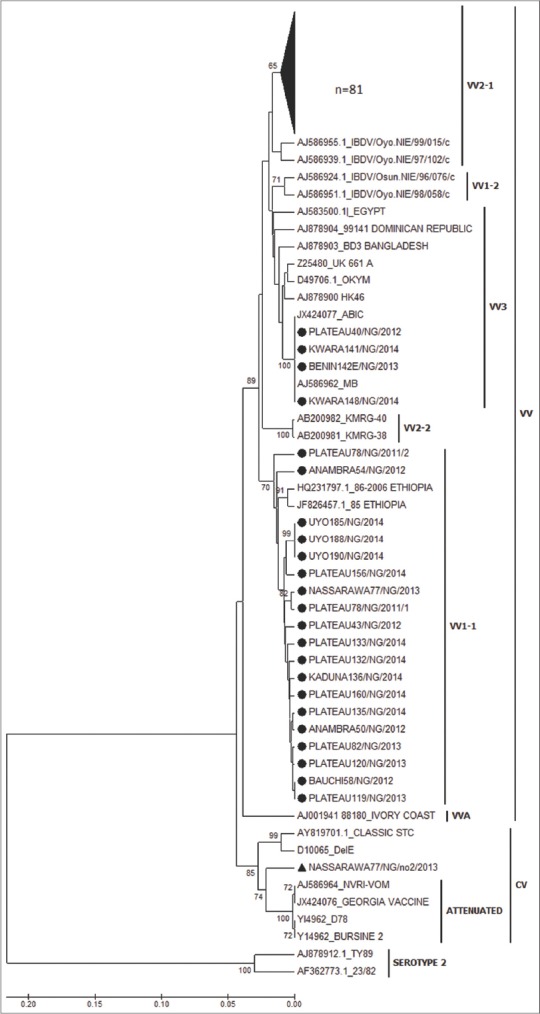
Phylogenetic analysis of Nigerian infectious bursal disease virus (IBDVs) and other IBDV strains from Genbank using unweighted pair-group methods using arithmetic averages with 1000 bootstrap replication.

All 105 Nigerian hvVP2 nucleotide sequences were included in the construction of NJ and UPGMA tree using Mega 6.0 [25], each with 1000 bootstrap replicates. Reference IBDV strains including known vvIBDV strains (UK661, Egypt, OKYM, and HK46), variant viruses (Del E and T1) and classic virus (STC) were used in the analysis. The phylogenetic tree showed separate groups for the vvIBDV, variant, vaccine and serotype 2 IBDV strains. Both the NJ and UPGMA trees (Figures-2 and 3) gave similar results. The Nigerian viruses were placed on branches within the vvIBDV group (bootstrap value 89% and 90%, respectively). One virus, Nassarawa77/NG/No2/2013, was in the non-vvIBDV cluster. It was also observed that the Nigerian viruses formed clusters of their own within the vvIBDV branches. The NJ tree reveals that 81 of the Nigerian viruses were closely related to vvIBDVs from the Dominican Republic and Bangladesh, 4 Nigerian viruses were most closely related to MB and ABIC vaccines, while the remaining 19 Nigerian viruses were closely related to vvIBDV strains from Ethiopia. Likewise, on the phylogenetic tree, 100 of the Nigerian viruses clustered with the African vv type while 4 clustered with the European/Asian vv types.

### Phylogenetic analysis of segment B

**Figure-4:**
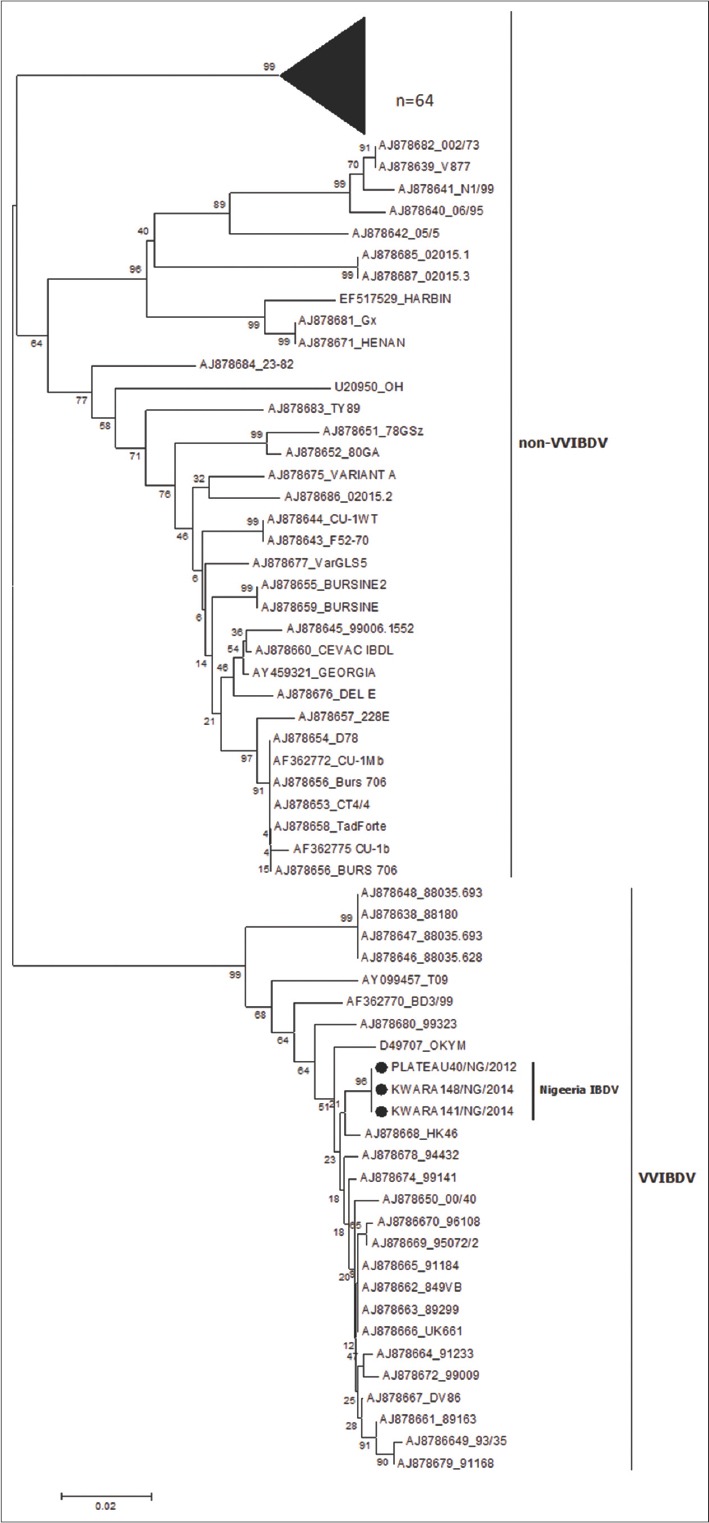
Phylogenetic analysis of the VP1 nucleotide sequences of Nigerian infectious bursal disease virus (IBDVs) and some IBDV VP1sequences from Genbank using the neighbor joining method with 1000 bootstrap replication.

An NJ phylogenetic tree was constructed with segment B nucleotide sequences from the 67 Nigerian viruses, IBDV reference strains [11] one turkey IBDV isolate from Nigeria and the OH serotype 2 strain from the US (Figure-4). The Nigerian viruses formed two lineages: 64 viruses grouped with non-vvIBDV strains but formed a unique cluster of their own while 3 viruses were found in a cluster within the vvIBDV branch. All 67 Nigerian viruses had a vvIBDV-like hvVP2 sequence. Therefore, the grouping of these 64 viruses with the non-vvIBDVs indicates they are probably reassortants.

## Discussion

In this study, we investigated reported suspected outbreaks of IBD in vaccinated and unvaccinated chicken flocks from different poultry farms in nine states in Nigeria during the years 2009-2014. The study was designed to determine the strain(s) of IBDV circulating within these states and the presence of reassortants. The vvIBDV strains have been reported in vaccinated and unvaccinated poultry flocks in some parts of Nigeria [17-21]. Vaccination failure could be due to changes in the hvVP2 of IBDV that may result from immunological pressure [12]. Although there are exceptions, the most vvIBDV exhibit unique genetic characteristics in the hvVP2 region at amino acid residues A222, I242, I256, I294 and S299 [22,26]. To accurately diagnose a vvIBDV phenotype, both genome segments A and B need to be analyzed [27]. In this study, the hvVP2 and a portion of the viral genome segment B were sequenced followed by phylogenetic analysis. Due to the import restriction on live viruses into the United States where this study was conducted, confirmation of the pathogenicity of the Nigerian viruses was not possible. Since genome reassorted viruses that contain a vvIBDV genome segment A and a non-vvIBDV genome segment B have shown reduced pathogenicity, the need for molecular classification of both genomes is required for a molecular diagnosis [11,13]. Nucleotide and amino acid sequences from the vv, variant and classic IBDV phenotypes [28] were used for comparison to the isolates circulating within Nigeria.

The deduced amino acid sequences of the hvVP2 region of the Nigerian viruses (aa 210-443) revealed that the majority of them had amino acids characteristic of vvIBDVs (A222, I242, I256, I294 and S299) [22,26]. However, some substitution mutations were observed. Two of the Nigerian viruses had an A222T mutation while 16 had a Q219T and 1 had a Q219P mutation (Table-1). Changes in the amino acid residues in hvVP2 occur frequently in the four hydrophilic loops thereby causing antigenic drift [4,29]. Some mutations in the Nigerian viruses could affect their antigenicity since they occurred on the first hydrophilic loop P_BC_ [4]. A substitution mutation G254S seen in all of the Nigerian viruses except four may also cause antigenic drift due to its location on the P_DE_ loop of VP2 [4]. This mutation (G254S) was found in previously published IBDV strains from northwestern Nigeria [21] but not in IBDV strains from southwestern Nigeria [20] indicating it may be geographically unique to those regions. Adamu *et al*. [21] noted that the non-synonymous mutations at amino acid position 254 and other synonymous mutations affected phylogenetic clustering of Nigerian and other IBDV strains and that different clusters of IBDV strains with identical VP2 amino acid sequences were formed based on a specific pattern of nucleotide mutations. These clusters provide clues to the evolution and spread of IBDV in Nigeria, but since their amino acids are identical, they would be expected to have similar antigenic phenotypes.

Phylogenetic analyses based on the hvVP2 amino acids (Figure-2) suggest that most of the Nigerian viruses belong to the vvIBDV group; only one virus was found in the non-vvIBDV cluster. This virus (Nassarawa77/NG/no2/2013) was detected from a flock that was vaccinated against IBDV. Incidentally, a Nigerian IBDV (Nassarawa77/NG/2013) that clustered with the vvIBDV was detected from the same sample. Coinfection with different genotypes of IBDV in the same bursa has been reported [30]. It was noted that the 81 Nigerian viruses formed a separate unique cluster and had amino acids S254, S299, and A300. Another four viruses that clustered within the vvIBDV were on a separate branch and had amino acids T272, N279 with S299 and E300. These viruses that formed a unique branch within the vvIBDV also included two IBDV vaccines (MB and ABIC) that also had amino acids T272 and N279. Field IBDVs that share similar sequences with vaccine IBDV strains have been reported [21,22]. Adamu *et al*. [21] implicated the indiscriminate use of IBDV vaccines from various countries in Nigeria as the source of spread of IBDV in the field. The remaining 19 Nigerian viruses that formed their own cluster were grouped on a branch that seems to be genetically divergent from the larger group. Although they had the amino acids typical of vvIBDVs, the mutations T219, P219, S269, and Q300 were observed. Comparison of the amino acid sequences of the viruses used in this study with previously published Nigerian IBDV isolates showed some similarity [20]. The only exceptions were substitution mutations seen at amino acid position T219 and S269 in some of the viruses used in this study but not seen in previously published Nigerian IBDV [18-21]. These mutations suggest that vvIBDV strains are continuing to evolve in Nigeria. The African vvIBD type are tentatively subdivided into VV1 which includes IBDV strains from Nigeria, Ethiopia and Zambia, and VV2 with IBDV strains from Nigeria, Tanzania and Zambia. The VV3 subgroup contains IBDV strains from Asia, Africa, Europe and other countries [31,32]. The hvVP2 sequences of the Nigerian viruses used were tentatively found in the VV1-1, VV2-1sub clusters and VV3 [19,30] (Figure-2). None of the previously characterized Nigerian IBDVs where seen in VV1-1 subcluster. Results obtained from the comparison of the VP1 region of the 67 Nigerian viruses with others from GenBank revealed the presence of reassortants; 64 viruses had vvIBDV hvVP2 sequences but non-vvIBDV VP1 amino acid sequences. The amino acid motifs observed at the triplet positions (145-147) of the Nigerian reassortant viruses were QEG, HEG, QDG, and HDG. The remaining three viruses had VP1 sequences that closely matched the vvIBDV, including TDN at the triplet position [10]. This observation is in agreement with other reports of reassortant strains of IBDV with a vvIBDV-like segment A and a non-vvIBDV like segment B [11]. These reassortants were found to be less pathogenic in specific-pathogen-free chickens. Pathogenicity studies of the Nigerian reassortant viruses would be necessary to determine the virulence of the reassorted viruses identified in our study.

In this study, we report IBDV with the genetic markers of vvIBDV circulating in nine states of Nigeria and also for the first time in the Nigerian states of Anambra, Akwa Ibom, Kwara, Nassarawa, and Edo. Only one IBDV detected in our study lacked the genetic markers of vvIBDV. We also report for the first time genetic reassortment between segment A and B of IBDVs in Nigeria with a segment A derived from vvIBDV and a segment B from non-vvIBDV-like viruses. Further study on the pathogenicity of these reassorted Nigerian IBDV strains is needed. There is also need to review the IBDV strains currently used for vaccine production in Nigeria as the use of live IBD vaccines has been implicated in the emergence of reassortant IBDV strains [33].

## Authors’ Contributions

ION designed the study, conducted the experimental work, analyzed and interpreted the data and drafted the manuscript. IS, CIN, GOE, FJCO, LOM and DJJ participated in the study design, analysis and interpretation of data and drafting of the manuscript. All authors read and approved the final manuscript.
